# Effects of Germination, Fermentation and Extrusion on the Nutritional, Cooking and Sensory Properties of Brown Rice Products: A Comparative Study

**DOI:** 10.3390/foods12071542

**Published:** 2023-04-05

**Authors:** Zhanqian Ma, Xiaotong Zhai, Na Zhang, Bin Tan

**Affiliations:** 1 School of Food Engineering, Harbin University of Commerce, Harbin 150076, China; 2 Academy of National Food and Strategic Reserves Administration, Beijing 100037, China

**Keywords:** germination, fermentation, extrusion, brown rice products, dietary fiber, texture properties, color

## Abstract

In this study, cooked brown rice (BR), germinated brown rice (GBR), fermented brown rice (FBR) and white rice (WR) were prepared by traditional cooking techniques, and extruded brown rice (EBR) was obtained by extrusion processing technology. The nutritional, cooking and sensory properties of different BR products were investigated. The results indicated that the soluble dietary fiber (SDF) content, free total phenolic content (TPC), total flavonoid content (TFC) and antioxidant capacity (DPPH, ABTS, T-AOC) in processed BR products were significantly higher than those in cooked BR and WR. The values of SDF, free TPC, TFC and T-AOC in EBR increased by 38.78%, 232.36%, 102.01% and 153.92%, respectively, compared with cooked BR. Cooked FBR and EBR had more nutrients, required less cooking time, had a softer texture and were whiter than cooked GBR and BR, especially EBR. In addition, the water absorption rate of EBR was 14.29% and 25.41% higher than that of cooked FBR and GBR. The hardness of EBR was significantly lower than that of cooked FBR and BR, even lower than that of cooked WR. However, there was no significant difference between the hardness of cooked GBR and that of cooked BR. The flavor compounds in EBR were similar to that of cooked WR, while those in cooked GBR and FBR did not differ greatly compared to cooked BR. Collectively, cooked FBR and EBR had better nutritional value, cooking and sensory properties than cooked BR, and the comprehensive value of EBR was higher.

## 1. Introduction

Rice is one of the world’s most important cereals, providing the main food for more than 50% of the world’s population [1]. Brown rice is a rice seed from which only the husk is dehulled, and it consists of the endosperm (92%), bran (5–6%) and germ (2–3%) [2]. Traditionally, rice is consumed as white rice, although the excessively refined milling processes of the production of white rice result in a large amount of food waste and nutrient loss. Studies have shown that rice bran is rich in bio-functional components which are beneficial for human health, including lipids, proteins, vitamins, fibers, polyphenols, etc. [3]. The regular consumption of brown rice products is associated with reduced risks of chronic diseases such as type II diabetes, cardiovascular disease and obesity [4]. The unique phytochemicals and dietary fiber that exist in rice bran contribute to these beneficial effects [1].

Normally, rice is consumed after cooking. Different processing methods will affect the nutrition and edible quality of rice [5]. Compared to white rice products, although brown rice has a higher nutritional value, the existence of the bran layer leads to some defects, including longer cooking time, higher hardness and chewiness of the products. These defects greatly reduce the sensory qualities of brown rice, further limiting its development and application in industry. Thus, it is necessary to find solutions for improving the eating qualities of brown rice based on retaining nutrients as much as possible.

Physical and biological techniques are applied as pretreatment methods to improve the quality of whole grains before they are cooked and consumed as whole grain foods. Common physical techniques include partial grinding, radiation, ultrasonic, etc. Based on previous studies, the physical techniques could alter the surface morphology of brown rice, then accelerate water penetration into the interior to improve the cooking and eating quality of brown rice products. Moreover, these physical techniques can also inactivate microorganisms and spoilage enzymes in grains, thus extending the shelf life of brown rice products. However, most of these techniques have complex processing processes as well as lead to nutrient loss. For example, Choi et al. [6] used a laboratory FS-2000 rice polisher to process the experimental samples and found that the content of total phenol, individual phenolic acid and anthocyanin in black rice decreased significantly with the increase in milling degree from 0 to 4.2% and 10.5%. UV-C irradiation (dosage of 6.18 kJ/cm^2^) for 3 h reduced the total phenolic content in black and red rice [7]. Ultrasound treatment caused the degradation of some phenolic compounds and vitamins, along with the change in color [8]. Extrusion is often used to enhance the nutritional properties and improve the texture of extruded products [9]. Wang et al. [2] reported that the total phenolic content, antioxidant capacity and texture properties (hardness, springiness and chewiness) of staple noodles made from wheat–buckwheat composite flour were significantly increased by adding extruded buckwheat flour with a water content of 30%.

Biological techniques used as pretreatment methods in whole grain food processing mainly include germination, fermentation and exogenous enzyme treatment, for improving the color and taste of products and preserving the nutritional properties of cereal foods. Wang et al. [10] found that germinated brown rice accumulated more nutrients and had a softer texture than brown rice. Compared with unfermented rice flour, rice noodles prepared from natural fermented rice flour showed a favorable chewy mouth-feel and nutritional value [11].

At present, most of the research focuses on the technical process optimization of these physical or biological pretreatment techniques, and their influence on the qualities of whole grain materials, respectively. The systematic comparative studies on the qualities of cooked brown rice products made from germinated, fermented or extruded brown rice are still limited; thus, relative study is necessary. The aim of the present study was to assess the nutritional (dietary fiber, phenolics, γ-oryzanol, GABA, antioxidant properties), cooking and sensory properties of cooked brown rice, white rice, germinated, fermented brown rice and extruded brown rice. This paper will provide new ideas and a basis for the selection of pretreatment or processing methods and the consumption of brown rice products with improved qualities.

## 2. Materials and Methods

### 2.1. Materials

Dao Huaxiang rice grains (Wuyoudao No.4, one variety of non-pigmented rice, long grain) were cultivated in the year 2021 in Wuchang district (Heilongjiang province, China). Brown rice is a rice grain prepared after removing the rice husks with a rice huller, including the cortex, aleurone layer and endosperm. The rice grain obtained after removing about 90% of the rice bran in the brown rice is white rice.

*Lactobacillus plantarum* (CICC No. 22696) was purchased from the China Industrial Culture Collection Center (Beijing, China). α-Amylase (enzyme activity [EA] ≥ 150,000 U/mL), amyloglucosidase (EA ≥ 110,000 U/g) and protease (EA ≥ 500,000 U/g) were supplied by Macklin Biochemical Co., Ltd. (Shanghai, China). Dietary fiber assay kit and antioxidant capacity assay kit (T-AOC) were purchased from Megazyme Co. (Bray, Wicklow, Ireland) and Jiancheng Bioengineering Institute (Nanjing, China). Other reagents used in this study were of analytical grade and originated from Chemical Co. (Beijing, China).

### 2.2. Preparation of Brown Rice Products

#### 2.2.1. Germination

The brown rice sample was washed and soaked in excessive warm water (1:5 *w*/*v*) for 12 h and allowed to germinate naturally for 54 h, at 30 °C, with 95% relative humidity in the germinator (N-01, C&R Machinery Technology Co., Nanjing, China). The germinated brown rice (GBR) sample was dried (HG-9055A Blast Drying Oven, Senxin Experimental Instrument Co., Ltd., Shanghai, China) at 50 °C for 12 h and then stored at −20 °C until analysis.

#### 2.2.2. Fermentation

According to the method of Liu et al. [12] with slight modifications. For inoculation, *L. plantarum* (1.0 × 10^9^ CFU/mL) was precultured in MRS broth in a rotary shaker (150 rpm) at 37 °C for 24 h to prepare liquid inoculum for the fermentation of brown rice (fermentation time: 24 h; fermentation temperature: 37 °C; inoculum concentration: 1%; and moisture content: 50%). The fermented brown rice (FBR) sample was dried (HG-9055A Blast Drying Oven, Senxin Experimental Instrument Co., Ltd., Shanghai, China) at 50 °C for 12 h and then stored at −20 °C until analysis.

#### 2.2.3. Cooking of Rice

Finally, the BR, GBR, FBR and WR samples were cooked (1:1.4, *w*/*v*) by a commercial SR-DE rice cooker (Panasonic Co., Osaka, Japan) in standard mode. The cooked rice samples were dried at 50 °C for 12 h (HG-9055A Blast Drying Oven, Senxin Experimental Instrument Co., Ltd., Shanghai, China). Finally, the cooked BR, GBR, FBR and WR were ground by a mechanical grinder (WF-20B, Keyi Machinery Equipment Co., Ltd., Nanjing, China), passed through a 100-mesh sieve, and then stored at −20 °C until analysis.

#### 2.2.4. Extrusion

The rice sample was extruded using a twin-screw extruder (FMHE-36, FUMACH, Changsha, China). The feed quantity of rice was 20 kg/h and the moisture content was adjusted to 24%. The barrel zone temperatures were set constant at 40, 110, 120, 80 and 70 °C, and the extrusion temperature and screw speed were 160 °C and 190 r/min, respectively. The extruded brown rice (EBR) sample was cooled to ambient temperature, ground by a mechanical grinder (WF-20B, Keyi Machinery Equipment Co., Ltd., Nanjing, China), passed through a 100-mesh sieve, and then stored at −20 °C.

### 2.3. Determination of Nutritional Properties

#### 2.3.1. Proximate Composition

The moisture, crude fat and ash contents were measured by the AOAC [13]. The crude protein content was analyzed using an Elementar rapid Ncube (Hanau, Germany) with a nitrogen conversion factor of 6.20. Total starch content was determined by a total starch kit (Megazyme, Wicklow, Ireland). Energy value was determined by a CA-HM Calorimeter (JWP, Tokyo, Japan).

#### 2.3.2. The Content of Bioactive Substances

The extraction of free and bound phenolic compounds and the total phenolic content (TPC) and total flavonoid content (TFC) of rice were measured according to our previous study [4]. The TPC of a sample was determined using the Folin–Ciocalteu colorimetric method. The TFC was measured by the NaNO_2_-Al(NO_3_) method. The contents of soluble dietary fiber (SDF) and insoluble dietary fiber (IDF) were determined by the enzymatic gravimetric method [14]. GABA and γ-oryzanol contents were determined by HPLC, as reported by Ding et al. [15] and Cho et al. [16].

#### 2.3.3. Antioxidant Properties

DPPH, ABTS free radical scavenging capacity and total antioxidant capacity (T-AOC) were determined by the method of Ma et al. [4].

DPPH radical scavenging activity assay. Briefly, 0.6 mL of appropriately diluted extract was combined with 3.0 mL of freshly made DPPH radical solution (0.1 mM/L, prepared in 95% methanol). The absorbance was determined at 517 nm after light avoidance reaction for 20 min. DPPH was reported as μmol of Trolox/100 g of dry weight.

ABTS radical scavenging activity assay. Briefly, 0.2 mL of appropriately diluted extract was combined with 3.0 mL of ABTS**·**^+^ solution. The mixed solution was protected from light for 6 min and the absorbance was measured at 734 nm. ABTS was reported as μmol of Trolox/100 g of dry weight.

The total antioxidant activity was determined according to the requirements of the total antioxidant capacity test kit provided by the Nanjing Institute of Bioengineering.

### 2.4. Determination of Cooking Properties

The water uptake ratio, cooking time and solid content of the brown rice samples were measured by the method of Kumar et al. [17] and Patil et al. [18]. The cooking properties of the BR, GBR and FBR samples were determined after cooking with a rice cooker. EBR was soaked in a certain volume of boiling water. After the EBR was completely rehydrated (the center of the grain was completely softened), the water was drained and the cooking properties were measured.

### 2.5. Determination of Sensory Properties

#### 2.5.1. Texture Properties

The texture characteristics of brown rice products were measured by the TPA (TA XT Plus, Stable Micro System, Surrey, UK) of a texture analyzer. In this method, 5.0 g of the cooked rice (BR, GBR, FBR) and EBR softened in boiling water samples were placed in a circular container and pressed to 40% of the sample thickness with a cylindrical probe at a speed of 1 mm/s. The measured interval time was 5 s, and the triggering force was 5.0 g. From the texture profiles, the values of hardness, adhesiveness, cohesiveness, springiness and chewiness were determined.

#### 2.5.2. Color

The color of brown rice products was measured in terms of L* value (lightness), a* value (redness) and b* (yellowness) using a CR-400 Chroma Meter (KMSA, Tokyo, Japan). The colorimeter was set to an illuminant condition D65 (medium daylight) and a 10° (field of view) standard observer. Parameter a* describes a red-green color (positive a* values indicate redness, negative a* values indicate greenness). Parameter b* describes a yellow-blue color (positive b* values indicate yellowness, negative b* values indicate blueness).

#### 2.5.3. Volatile Flavor Substances

The fast GC e-nose called Heracles II (Alpha M.O.S., Toulouse, France) was used for the analysis. It consisted of a sampling system (HS100 autosampler), a detector system, and a data acquisition and processing system (Alpha MOS proprietary software (Alpha Soft, Toulouse, France)). For the identification of volatile compounds, the alkane standard solution was used.

### 2.6. Statistical Analyses

Data were analyzed by a one-way analysis of variance (ANOVA), using SPSS 17.0 (SPSS Inc. Chicago, IL, USA). Significant differences were determined using Duncan’s multiple range test. Spearman’s correlation analysis was applied using GraphPad Prism 8.0 software (GraphPad Software Inc. San Diego, CA, USA). The significance levels were set at *p* < 0.05.

## 3. Results and Discussion

### 3.1. Proximate Compositions of Brown Rice Products

The proximate compositions of cooked BR, WR, GBR, FBR and EBR are shown in Table 1. The major components of cooked BR, WR, GBR, FBR and EBR were moisture, fat, starch, protein and ash. The contents of fat, protein and ash in cooked BR, GBR, FBR and EBR were significantly higher than those in cooked WR (*p <* 0.05). This is similar to the previous studies of Wang et al. [10], who found that rice bran contains most of the nutrients in rice, including protein, fat, dietary fiber, phenolic compounds, vitamins, etc. Compared with cooked BR, germination, fermentation and extrusion significantly increased the levels of protein (*p <* 0.05). The protein values of cooked GBR, FBR and EBR were 1.84%, 5.26% and 20.32% higher than that of cooked BR, respectively. This may be related to the increase in protease activity [19]. The three processed BR products showed lower starch content, especially EBR. The starch chain is more easily broken by extrusion, resulting in the degradation of starch and the formation of small oligosaccharides [20]. In addition, there was no significant difference in energy between cooked GBR, FBR and BR, while the energy of EBR was 3.46% lower than that of cooked BR.

### 3.2. Content of Bioactive Components in Brown Rice Products

#### 3.2.1. Dietary Fiber (DF)

The DF contents of brown rice products are presented in Table 2. The SDF and IDF contents of cooked WR were significantly lower than that of cooked BR products (*p <* 0.05). Varying processing techniques have different effects on dietary fiber. Most studies proved that an increase in SDF content was the main trend for grains upon germination [19,21]. The present study is in accordance with the above results. Compared with cooked BR, the SDF concentration of cooked GBR increased by 21.43%, which may be due to a drop in galactan content during germination [22]. On the contrary, several studies have shown that the germination of BR affords reduced levels of SDF [23]. The diminished content of SDF could be attributed to the breakdown and utilization by the growing sprouts. Similarly, the effects of fermentation and extrusion on the SDF content of brown rice were similar to that of germination. The SDF contents of cooked FBR and EBR increased by 24.49% and 38.78%, respectively, compared with cooked BR. The increase in SDF content in EBR was due to the high shear force that promoted the dissolution of dietary fiber from the bran layer [24]. Although there was no significant difference in IDF content among all processed BR products, the IDF content in cooked FBR was lower than that in cooked BR. This was related to the degradation of IDF into SDF by active enzymes produced during fermentation [25]. In general, the extrusion and fermentation treatment on DF appeared to be more effective than germination, and the extrusion effect was the best.

#### 3.2.2. Total Phenolic Content (TPC) and Total Flavonoid Content (TFC)

As shown in Table 2, the TPCs of free and bound fractions in cooked BR, GBR and FBR were much higher than those in cooked WR (*p <* 0.05). The TPCs of free fractions in cooked GBR, FBR and EBR were obviously increased, by 29.54%, 9.98% and 232.36%, respectively. The increment of TPC in cooked GBR due to the activation of cell wall-degrading enzymes and phenylalanine ammonia lyase upon germination resulted in the release and biosynthesis of phenolic compounds [21]. In addition, the breakdown of cell wall structure caused by fermentation also leads to the synthesis of phenolic compounds [26]. However, the TPCs of bound fractions in cooked GBR were the highest, while in EBR the lowest. Compared with cooked BR, the TPC of the bound fractions of EBR decreased by 82.58%. These results demonstrated that germination, fermentation and extrusion all had a positive influence on the TPC of brown rice, especially extrusion, which significantly increased the free TPC and decreased the bound TPC (*p <* 0.05). These results are in accordance with those of Liu et al. [27] and Ortiz-Cruz et al. [9]. The significant increase in free TPC in EBR was probably due to the bound phenolic compounds being converted into polyphenols in free form arising from the shearing effect of extrusion [9].

Table 2 shows the distribution of TFC for free and bound fractions from different BR products. The free and bound TFCs of cooked FBR and EBR were significantly higher than those of cooked BR (*p <* 0.05). Compared with cooked BR, the free TFC of cooked FBR and EBR increased by 20.65% and 102.01%, while the bound TFC increased by 153.77% and 108.24%, respectively. The change of TFC was likely due to the activation of different enzymatic systems during processing, resulting in phenolic biosynthesis [21]. Compared with cooked BR, the bound TFC of cooked GBR decreased by 36.97%. Compared with cooked BR, the content of free TFC increased, while the content of bound TFC decreased in cooked GBR. This can be explained by the hydrolysis of cell wall-bound polyphenol compounds. In addition to this, the degree of increase of free TFC in EBR was significantly higher than that of cooked GBR (*p <* 0.05). Ortiz-Cruz et al. [9] concluded that this significant increase was probably due to the dissociation of bound form polyphenol arising from the shearing effect of extrusion.

#### 3.2.3. GABA

γ-Amino butyric acid (GABA) is a free modified amino acid that helps to inhibit cancer cell proliferation and reduces blood pressure. The GABA contents of cooked GBR and FBR were significantly higher than that of cooked BR (*p <* 0.05), and the comparative increase in cooked GBR (192.46%) was higher as compared to cooked FBR (27.94%) (Table 2). Compared with cooked BR, the GABA content of EBR decreased by 33.04%. Earlier researchers [28,29] reported an increase in GABA content as one of the nutritional benefits of germination and fermentation processes. GABA has been rapidly produced in response to anaerobic conditions, low pH, low or high temperatures and darkness [30]. In particular, the enzyme systems associated with endogenous enzymes and glutamate decarboxylase are activated, while major components such as starch and proteins may be degraded during germination, resulting in some secondary metabolites such as GABA [31].

#### 3.2.4. γ-Oryzanol

Among the three kinds of BR products, only the γ-oryzanol content in cooked FBR was 1.72% higher than that in cooked BR (Table 2). This effect could be attributed to the loose particles and increased void space of brown rice after high pressure sterilization, which was conducive to the growth and metabolism of *Lactobacillus plantarum*, thus promoting the enrichment of γ-oryzanol [32]. Moreover, our results are consistent with studies showing a decreased γ-oryzanol concentration in cooked GBR [33,34]. Compared with cooked BR, the content of γ-oryzanol in GBR decreased by 6.23%, respectively. The cause might be related to the fact that germination increased feruloyl esterase activity involved in the hydrolysis of esters of phenolic acids, such as γ-oryzanol (esters of trans-ferulic acid) [35]. Similarly, the γ-oryzanol content of EBR decreased by 48.43%, showing a significant decrease (*p <* 0.05). The physical force of the extruder damaged the cell membrane where the γ-oryzanol was present, and γ-oryzanol in damaged particles may be more easily oxidized [25]. γ-oryzanol has previously been shown to display various biological activities, including antioxidant, anti-inflammatory and anti-tumor activities [36]. In addition, γ-oryzanol has also been reported to reduce blood cholesterol levels in humans and hamsters [16].

### 3.3. Antioxidant Properties of Brown Rice Products

**Figure 1:**
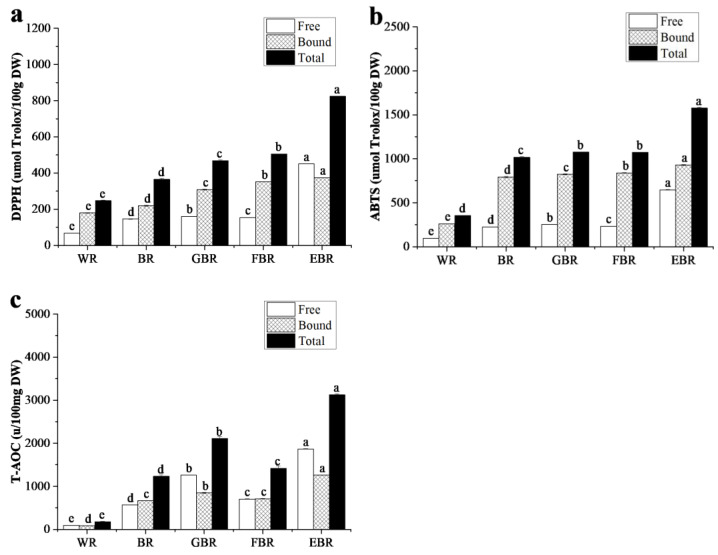
Antioxidant activity of different brown rice products (*n* = 3). (**a**) DPPH free radical scavenging capacity. (**b**) ABTS free radical scavenging capacity. (**c**) Total antioxidant capacity (T-AOC). The data columns are mean ± standard deviation from four independent experiments (WR—white rice, BR—brown rice, GBR—germinated brown rice, FBR—fermented brown rice, EBR—extruded brown rice). Different lowercase letters indicate significant differences among processing techniques (*p* < 0.05). DW: dry basis weight.

Foods rich in antioxidants have beneficial health effects, such as reducing the risk of non-communicable diseases and premature ageing [37]. The DPPH, ABTS free radical scavenging capacity and antioxidant capacity (T-AOC) of different brown rice products are shown in Figure 1. The DPPH, ABTS free radical scavenging and T-AOC activities of free, bound and total phenolic extracted from cooked GBR, FBR and EBR were significantly higher than those of cooked BR (*p* < 0.05) (Figure 1a–c), and the extent of increase in the EBR sample was higher. Compared with cooked BR, the values of total T-AOC activity in cooked GBR, FBR and EBR increased by 71.24%, 15.00% and 153.92%, respectively. Moreover, significant differences in DPPH**·**, ABTS**·** and T-AOC values were found among the three processed BR products (*p* < 0.05). The results of this work show significant DPPH and ABTS free radical inhibition ability for cooked GBR and FBR, which is consistent with data reported by Hiran et al. [33]. These results are also in agreement with data in the literature reported by Sharma et al. [31] and Purewal et al. [38], who noticed an increase in the DPPH free radical scavenging activity of millet by 77.96% and 251.92% after germination and fermentation. When the endosperm is modified by hydrolase, some bound components may be released during germination and play a role in scavenging DPPH free radicals [31]. Moreover, the antioxidant activity of whole grain rice was closely correlated with phenolics contents [4]. In Table 2, the phenolic contents of three processed BR products were significantly higher than that of cooked BR (*p* < 0.05). Therefore, the antioxidant activities of cooked GBR, FBR and EBR were mainly related to the increase in phenolic content. However, different test methods have different evaluation mechanisms of antioxidant activity. Thus, the comprehensive evaluation of antioxidant activity of rice can be combined with various antioxidant determination methods.

### 3.4. Correlation Analysis between Bioactive Substances and Antioxidant Activity

**Figure 2:**
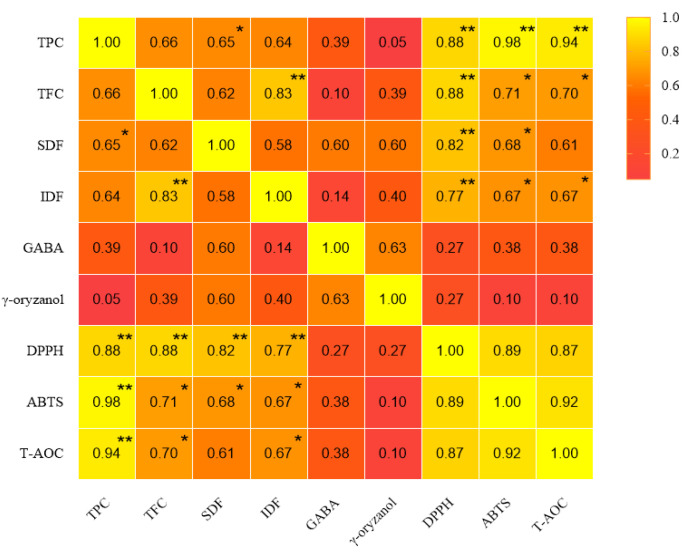
Correlation between the contents of bioactive substances and antioxidant activity of different brown rice products (*n* = 3). * *p* < 0.05, ** *p* < 0.01, ns = not significant.

Brown rice is considered a grain that contains several potentially valuable phytochemical fractions, such as phenolic compounds, dietary fiber, GABA and γ-oryzanol, which are closely related to antioxidant or free radical scavenging capacity [16]. The correlation between the contents of TPC, TFC, SDF, IDF, GABA, γ-oryzanol and the antioxidant capacity of brown rice products is shown in Figure 2. Strong positive correlations (r^2^ = 0.88, 0.98 and 0.94, respectively) were found between TPC with DPPH, ABTS free radical inhibition ability and T-AOC, respectively. TFCs were also positively correlated with three antioxidant capacities (r^2^ = 0.88, 0.71 and 0.70, respectively). Therefore, it can be concluded that the contents of TPC and TFC were attributed to the antioxidant capacity of brown rice products. The results are consistent with the findings of Aguilar-Garcia et al. [39] and Chmiel et al. [26], who also reported a strong correlation between total phenolic content and antioxidant activity of rice bran.

Dietary fiber with strong antioxidant properties can improve the shelf-life of foods [40]. Furthermore, SDF has special physiological functions related to human health, such as the prevention of diabetes, cardiovascular diseases, colorectal cancer, etc. These physiological functions may be related to the antioxidant activity of SDF [41]. Dong et al. [42] found that the antioxidant activity of SDF was stronger than IDF, especially in removing free radicals. In the present study, SDF was positively correlated with DPPH, and ABTS free radical inhibition ability (r^2^ = 0.82 and 0.68, respectively). There was no significant correlation between the contents of GABA, γ-oryzanol and the antioxidant capacity. In the present study, the result of the free radical scavenging activity was in accordance with the phenolic, flavonoid and dietary fiber contents, which suggested that phenolics, flavonoids and dietary fiber were potent antioxidants and free radical scavengers.

### 3.5. Cooking Properties of Brown Rice Products

The cooking properties of rice samples, which can indicate the relationship between the water adsorption and eating quality of brown rice, are shown in Table 3. The adsorption of water by brown rice reflects the penetration of water into the rice during the soaking process. In order to ensure the rapid and complete gelatinization of starch, water should quickly penetrate into the core of rice grains during cooking. The better the water penetration, the shorter the cooking time and the softer the cooked BR [43,44,45]. According to the results, the water adsorption rate of cooked BR was 1.61%, which was significantly lower than those of cooked GBR, FBR and EBR (which were 1.85%, 2.03% and 2.32%, respectively) (*p* < 0.05). The cooking times of cooked GBR, FBR and EBR were 1.4, 1.5 and 9.5 times shorter than cooked BR. It can be seen that the water absorption rates of cooked GBR, FBR and EBR were significantly increased, whereas the cooking times were shortened (*p* < 0.05). These findings are consistent with Patil et al. [18] and Lu et al. [11], who stated that the germination and fermentation process caused cracks on the surface of brown rice, making it easier for moisture to enter the grain during cooking and softening the texture. Furthermore, Li et al. [46] found that the increase in heating temperature promoted the gelatinization of brown rice starch. The high temperature treatment in the extrusion process caused the starch of brown rice to become partially gelatinized, which could then accelerate the gelatinization during cooking and in turn decrease the cooking time. Therefore, it made the EBR easier to cook. When cooked, EBR showed greater water uptake and volume expansion compared with those of the control (i.e., BR).

In addition, Table 3 shows that different processed BR products have significant differences in the loss of solid content after cooking (*p* < 0.05). The solid contents of cooked WR, BR, GBR, FBR and EBR were 0.23, 0.05, 0.07, 0.09 and 0.11 g, respectively. The results showed that the solid content in cooked WR was higher than in BR products. This might be related to the lack of protection of the bran layer in cooked WR, which led to the high residual solid content in cooked rice soup. Compared with cooked BR, the increase in the solid contents of the three processed BR products was due to the increase in water absorption after cooking again. Higher water absorption increased the gelatinization and decomposition of starch, resulting in the further cooking loss of BR products [18,47]. The present results demonstrated that the cooking quality of cooked GBR, FBR and EBR could be improved by increasing water uptake ratio during the cooking process, especially the cooking quality of EBR.

### 3.6. Texture Properties of Brown Rice Products

Hardness is the force required to deform a material and is often considered a reflection of the general texture quality of the rice. The hardness of brown rice products varied depending on the processing technology (Table 4). The hardness values of cooked GBR, FBR and EBR were lower than that of cooked BR. Compared with cooked BR, the hardness values of cooked GBR, FBR and EBR were reduced by 0.51%, 33.87% and 81.86%, respectively. According to the results, fermentation and extrusion significantly reduced the hardness of brown rice products. The hardness of cooked FBR was close to that of cooked WR, and the hardness of EBR was the lowest. The reason for the decrease in the hardness of cooked FBR after cooking may be that fermentation softens the starch endosperm structure, allowing water to spread more easily into the kernel. The increase in water absorption resulted in the softening of the texture of cooked FBR [46]. This was also observed in the study of the water absorption rate (Table 3). Moreover, the high-temperature and high-heat conditions in the extrusion process caused the rice grains to expand quickly and increased the pore size, leading to the increase in water absorption [46,47]. In addition, the application of pressure resulted in a softer texture than other techniques or even white rice. Springiness refers to the degree to which a food returns to its original shape after the omission of the deforming force in the texture profile analysis of the product. According to Table 4, there was a significant difference among the springiness rates of GBR, FBR and EBR (*p* < 0.05). The maximum and minimum springiness rates were observed in cooked FBR and BR. This is because of the higher water adsorption capacity of cooked FBR and its more continuous structure in the treatments, which increased their viscosity and elasticity [47]. Adhesiveness is the degree to which the kernels adhere to contacting substances. Compared with cooked BR, the adhesiveness values of cooked GBR, FBR and EBR were improved sequentially; this was associated with the increased degree of hydration of starch granules [46]. A similar increment was also found in the cohesiveness of cooked GBR, FBR and EBR. Moreover, the chewiness values of cooked BR, GBR, FBR and EBR were significantly different (*p* < 0.05). Chewiness is a comprehensive presentation of the above-mentioned three indices of hardness, cohesiveness and springiness, meaning the energy necessary to chew rice for swallowing [2]. The chewiness values of cooked GBR, FBR and EBR were significantly lower than that of cooked BR, indicating that three processed BR products had higher eating quality than cooked BR. To sum up, it is interesting that cooked FBR and EBR have a better eating quality, which is almost similar to cooked WR.

### 3.7. Color Values of Brown Rice Products

The color values of cooked WR, BR, GBR, FBR and EBR are shown in Table 4. Considerably higher (*p* < 0.05) lightness (L* value) of cooked WR was observed together with lower (*p* < 0.05) yellowness (b* value) and redness (a* value) when compared to the color of the BR products. The color values (L*, a*, b*) of three processed BR products were significantly different from those of cooked BR. The whiteness of cooked GBR was lower than that of cooked BR; it implied that BR was whitened by germination treatment [18]. On the contrary, cooked FBR and EBR were whiter than cooked BR. The redness values (a* value) of cooked GBR, FBR and EBR were significantly higher (*p* < 0.05) than the color of cooked BR. Compared with cooked GBR and FBR, EBR had lower redness. For cooked products, FBR and BR had almost the same yellow values, but GBR was significantly more yellow than BR. Changes in the a* and b* values of the BR products were mainly caused by Maillard-type non-enzymatic browning [47].

### 3.8. Volatile Flavor Compounds of Brown Rice Products

**Figure 3:**
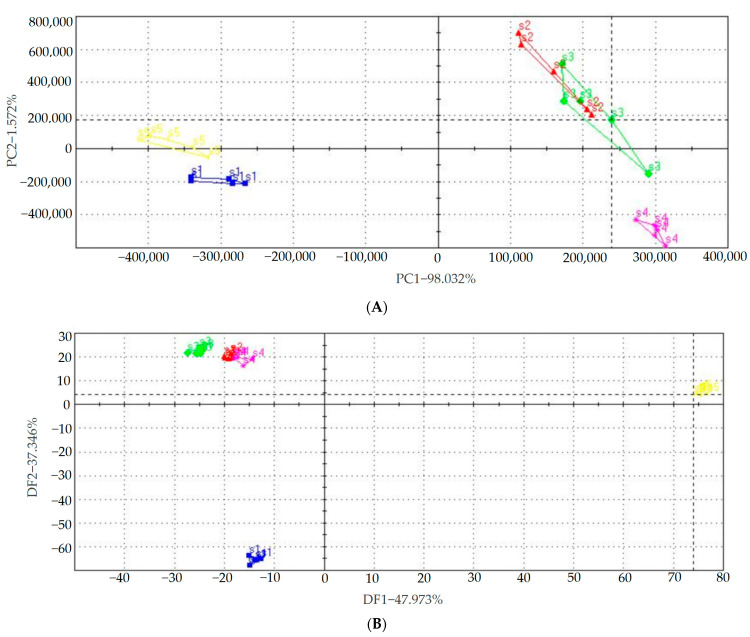
E-nose analysis results of different brown rice products (*n* = 5). (**A**) PCA score plot. (**B**) DFA score plot. Where S1: WR, S2: BR, S3: GBR, S4: FBR, S5: EBR (WR—white rice, BR—brown rice, GBR—germinated brown rice, FBR—fermented brown rice, EBR—extruded brown rice).

Figure 3A presents PCA score plots based on the peak area values for the discrimination of the volatile flavor compounds of different BR products. PC1 explains 98.03% of the total variance, and PC2 contributes 1.57% of the total variation. The first two PCs cumulatively represent 99.60% of the data variance, which appeared to provide sufficient information to explain the odor difference of BR products. The discrimination index was 85%, which indicates how distant each cluster is from the other [48]. Based on the distance between each sample cluster, the samples are divided into five groups in Figure 3A. The cooked BR, GBR and FBR samples were situated on the first and fourth quadrants of the map, whereas the cooked WR and EBR samples were placed on the second and third quadrants of the PCA map. This indicated that the volatile gases of cooked BR, GBR and FBR were different from those of cooked WR, while the volatile gases of EBR were close to that of cooked WR. Moreover, the volatile gases of cooked GBR and FBR did not change significantly compared with that of the cooked BR samples. Thus, the data that the e-nose extracted were similar to each other.

As shown in Figure 3B, similar to the PCA result, the discrimination was confirmed as all samples from the same BR products were grouped together and there was no intersection between the various groups analyzed. These discriminated groups correctly correspond to different BR products. The first discriminant function (DF1) explains 47.97% of the total variation, and the second discriminant function (DF2) explains 37.34% of the total variation. The total variation contributed by the two discriminant functions is 85.31%, which means these two variation sources reflect 85.31% of the original information. In Figure 3B, differences between individual samples belonging to the same processed BR products disappear.

The qualitative results in Table 5 identify 11 classes of volatile flavor substances, including hydrocarbons, alcohols, esters, aldehydes, furans, ketones, acids and so on. Aldehydes, alcohols, ketones and esters were the main volatile flavor components in brown rice, which is consistent with the research results of Sun et al. [49]. From the observation of Table 5, it is possible to highlight some major findings: some components are common to all BR products, as in the case of 1-propanol, 2-mercaptoethanol, acetaldehyde, propenal, 2-methylpropanal and butanal. The species of flavor substances identified in cooked GBR and FBR did not differ greatly compared to cooked BR, which were consistent with their close distances in PCA and DFA. Compared with cooked GBR and FBR, some other compounds were only detected in EBR, such as isopropyl acetate, 2-butanol and 1-propanethiol. These volatile substances endowed EBR with a strong fragrance and sweet taste [50,51]. In addition, the disappearance of furan and 2-methylfuran in EBR was also noted. It has been reported that furan is a cause of off-flavor in soybean oil [51].

## 4. Conclusions

In conclusion, the nutritional, cooking and sensory properties of brown rice products could be enhanced by fermentation and extrusion, but especially the latter. The contents of the SDF, free TPC, TFC and antioxidant activities of three processed BR products were significantly higher than those of cooked BR, and EBR was the highest, followed by cooked FBR and GBR (*p <* 0.05). Cooked FBR and EBR had a softer texture than cooked BR and GBR; thus, they could be chewed easier. EBR was easier to cook and required less cooking time. In view of the texture and cooking properties of BR products, extrusion could improve the elasticity and water absorption of BR, while the hardness and chewiness of EBR were also reduced compared to BR. In addition, cooked FBR and EBR were whiter than cooked BR and GBR, especially EBR. The flavor compounds in EBR were similar to cooked WR. This study could provide theoretical guidance for the selection of pretreatment or processing methods of brown rice products with improved qualities. However, the present study was only based on a single variety and type of rice, and its conclusions are limited. In the future, a wider range of rice samples (more genotypes/type of rice) should be analyzed, which has important implications for promoting the consumption of whole grain brown rice.

## Figures and Tables

**Table 1:** Basic components in different brown rice products (g/100g dry weight, DW).

Samples	Moisture	Fat	Starch	Protein	Ash	Energy (kcal)
WR	8.89 ± 0.01 ^b^	0.00 ± 0.00 ^d^	78.46 ± 2.56 ^a^	7.5 ± 0.06 ^c^	0.28 ± 0.00 ^c^	196.66 ± 3.80 ^b^
BR	8.33 ± 0.06 ^c^	0.08 ± 0.00 ^c^	70.09 ± 0.14 ^b^	8.17 ± 0.25 ^bc^	1.51 ± 0.01 ^a^	202.36 ± 1.17 ^a^
GBR	7.08 ± 0.02 ^e^	0.09 ± 0.00 ^c^	68.83 ± 0.08 ^b^	8.32 ± 0.56 ^bc^	1.18 ± 0.01 ^b^	203.83 ± 1.00 ^a^
FBR	7.51 ± 0.01 ^d^	0.14 ± 0.01 ^b^	68.08 ± 0.41 ^b^	8.60 ± 0.18 ^b^	1.48 ± 0.13 ^a^	201.16 ± 0.28 ^a^
EBR	13.16 ± 0.03 ^a^	0.15 ± 0.00 ^a^	61.63 ± 0.31 ^c^	9.83 ± 0.31 ^a^	1.59 ± 0.01 ^a^	195.35 ± 0.28 ^b^

Values are presented as mean ± standard deviation (*n* = 3). Different lowercase letters in each column indicate significant differences among processing techniques (*p* < 0.05).

**Table 2:** The content of bioactive components in different brown rice products.

Samples	Dietary Fiber (g/100g DW)		TPC (mg GAE/100 g DW)		TFC (mg RE/100 g DW)		GABA (mg/100g)	γ-Oryzanol (μg/g)
	SDF	IDF	Free Fraction	Bound Fraction	Free Fraction	Bound Fraction		
WR	0.96 ± 0.01 ^d^	1.88 ± 0.13 ^b^	2.84 ± 0.04 ^e^	3.09 ± 0.06 ^e^	27.88 ± 0.13 ^e^	20.60 ± 0.66 ^e^	0.65 ± 0.04 ^e^	0.67 ± 0.00 ^d^
BR	1.96 ± 0.05 ^c^	6.14 ± 0.13 ^a^	25.56 ± 0.75 ^d^	35.94 ± 0.44 ^b^	67.66 ± 1.43 ^d^	41.03 ± 0.30 ^c^	4.51 ± 0.10 ^c^	665.01 ± 15.34 ^a^
GBR	2.38 ± 0.19 ^b^	5.98 ± 0.33 ^a^	33.11 ± 0.07 ^b^	39.55 ± 0.06 ^a^	76.22 ± 1.41 ^c^	25.86 ± 0.04 ^d^	13.19 ± 0.18 ^a^	623.56 ± 13.28 ^b^
FBR	2.44 ± 0.03 ^b^	6.09 ± 0.29 ^a^	28.11 ± 0.25 ^c^	35.09 ± 0.17 ^c^	81.63 ± 0.87 ^b^	104.12 ± 1.24 ^a^	5.77 ± 0.09 ^b^	676.43 ± 12.47 ^a^
EBR	2.72 ± 0.02 ^a^	6.54 ± 0.37 ^a^	84.95 ± 0.53 ^a^	6.26 ± 0.02 ^d^	136.68 ± 0.02 ^a^	85.44 ± 0.62 ^b^	3.02 ± 0.12 ^d^	342.93 ± 0.71 ^c^

Values are presented as mean ± standard deviation (*n* = 3). Different lowercase letters in each column indicate significant differences among processing techniques (*p* < 0.05). SDF: soluble dietary fiber. IDF: insoluble dietary fiber. TPC: total phenolic content. TFC: total flavonoid content. GAE: gallic acid equivalent. RE: rutin equivalent. DW: dry basis weight.

**Table 3:** Cooking properties of different brown rice products.

Samples	Water Absorption Rate (%)	Cooking Time (min)	Solid Content (g/100 g DW)
WR	4.10 ± 0.13 ^a^	15.43 ± 0.35 ^d^	0.23 ± 0.02 ^a^
BR	1.61 ± 0.02 ^d^	25.83 ± 0.76 ^a^	0.05 ± 0.00 ^e^
GBR	1.85 ± 0.05 ^c^	18.80 ± 0.36 ^b^	0.07 ± 0.00 ^d^
FBR	2.03 ± 0.03 ^c^	17.63 ± 0.21 ^c^	0.09 ± 0.00 ^c^
EBR	2.32 ± 0.22 ^b^	2.73 ± 0.21 ^e^	0.11 ± 0.00 ^b^

Values are presented as mean ± standard deviation (*n* = 3). Different lowercase letters in each column indicate significant differences among processing techniques (*p* < 0.05).

**Table 4:** Texture properties and colors of different brown rice products.

Samples	Hardness/g	Adhesiveness	Springiness	Cohesiveness	Chewiness	L*	a*	b*
WR	1033 ± 64.89 ^c^	71.94 ± 3.20 ^c^	0.89 ± 0.05 ^ab^	0.57 ± 0.02 ^b^	519.9 ± 36.07 ^c^	65.81 ± 0.09 ^a^	0.50 ± 0.03 ^e^	5.96 ± 0.01 ^e^
BR	1876 ± 0.68 ^a^	15.12 ± 0.63 ^a^	0.71 ± 0.03 ^c^	0.43 ± 0.00 ^c^	788.2 ± 1.14 ^a^	54.64 ± 1.52 ^d^	0.59 ± 0.02 ^d^	10.15 ± 0.17 ^b^
GBR	1866 ± 44.88 ^a^	16.73 ± 0.65 ^a^	0.81 ± 0.04 ^b^	0.44 ± 0.01 ^c^	671.2 ± 25.83 ^b^	51.53 ± 0.05 ^e^	0.83 ± 0.02 ^b^	10.75 ± 0.03 ^a^
FBR	1240 ± 48.37 ^b^	18.01 ± 0.44 ^a^	0.93 ± 0.04 ^a^	0.46 ± 0.01 ^c^	358.9 ± 23.74 ^d^	56.29 ± 0.10 ^c^	0.88 ± 0.02 ^a^	9.38 ± 0.01 ^c^
EBR	340.3 ± 20.79 ^d^	28.42 ± 1.71 ^b^	0.86 ± 0.02 ^ab^	0.63 ± 0.02 ^a^	170.1 ± 12.71 ^e^	58.41 ± 0.19 ^b^	0.62 ± 0.04 ^c^	9.22 ± 0.03 ^d^

Values are presented as mean ± standard deviation (*n* = 3). Different lowercase letters in each column indicate significant differences among processing techniques (*p* < 0.05).

**Table 5:** Key volatile flavor compounds of different brown rice products.

Name of the Compound	Formula	Structure	Retention Time (s)	Samples				
				WR	BR	GBR	FBR	EBR
Methyl formate	C_2_H_4_O_2_	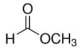	15.90	√	√	√	-	-
Vinyl acetate	C_4_H_6_O_2_	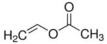	23.69	√	√	√	√	-
Methyl propanoate	C_4_H_8_O_2_	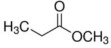	28.91	√	√	-	-	-
Isopropyl acetate	C_5_H_10_O_2_	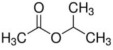	32.28	√	-	-	-	√
Methyl isobutyrate	C_5_H_10_O_2_	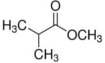	36.33	√	-	-	-	-
Ethyl propanoate	C_5_H_10_O_2_	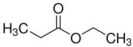	38.87	√	-	-	-	-
Methanethiol	CH_4_S	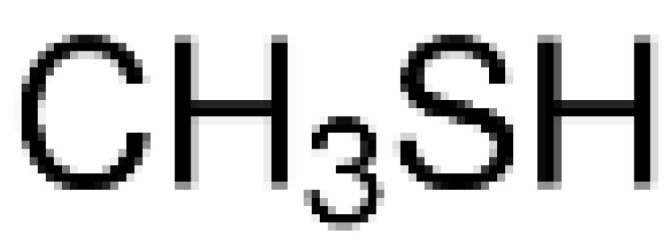	17.20	-	-	-	√	√
1-Propanol	C_3_H_8_O	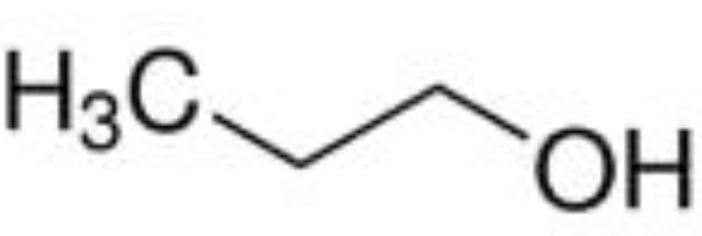	21.33	√	√	√	√	√
2-mercaptoethanol	C_2_H_6_O_S_	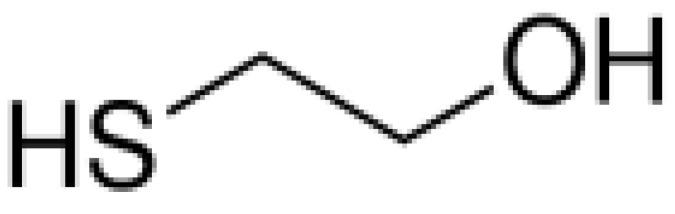	23.69	√	√	√	√	√
2-butanol	C_4_H_10_O	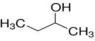	26.26	√	√	-	-	√
1-propanethiol	C_3_H_8_S	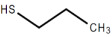	29.17	√	-	-	-	√
n-butanol	C_4_H_10_O	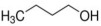	32.28	√	-	-	-	-
Trometamol	C_4_H_11_NO_3_	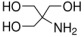	34.12	√	-	-	-	-
Acetaldehyde	C_2_H_4_O	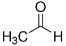	17.10	√	√	√	√	√
Propenal	C_3_H_4_O	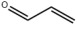	17.10	√	√	√	√	√
2-methylpropanal	C_4_H_8_O	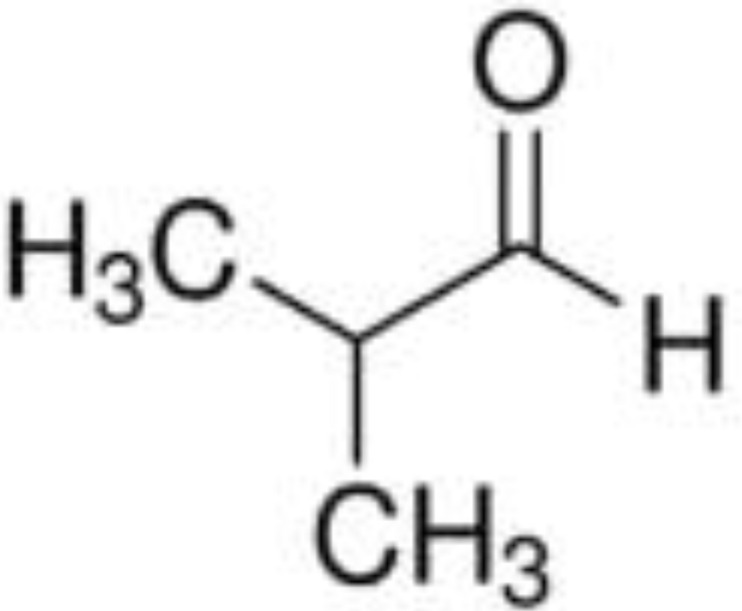	21.33	√	√	√	√	√
Butanal	C_4_H_8_O	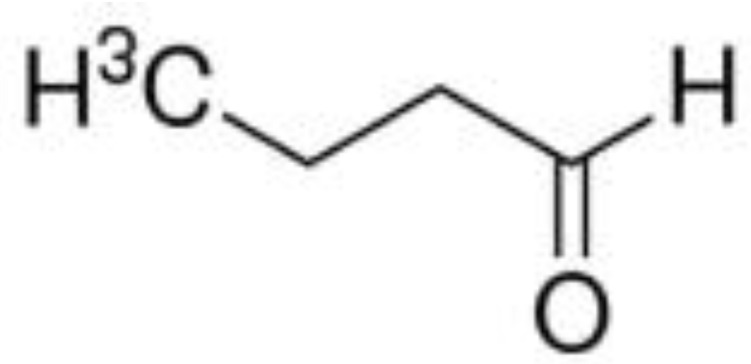	23.69	√	√	√	√	√
3-methylbutanal	C_5_H_10_O	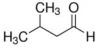	32.28	√	-	-	-	-
3-Pentanone	C_5_H_10_O	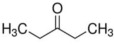	36.33	√	-	-	-	-
Acetoin	C_4_H_8_O_2_	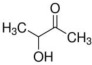	36.33	√	-	-	-	-
Furan	C_4_H_4_O	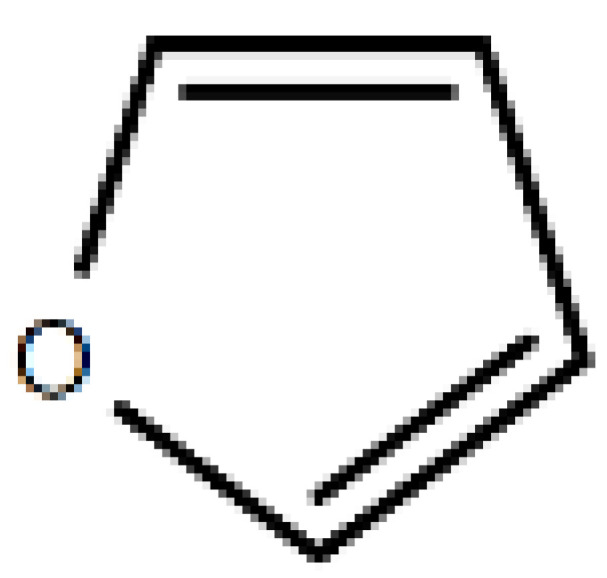	20.83	√	√	√	√	-
2-methylfuran	C_5_H_6_O	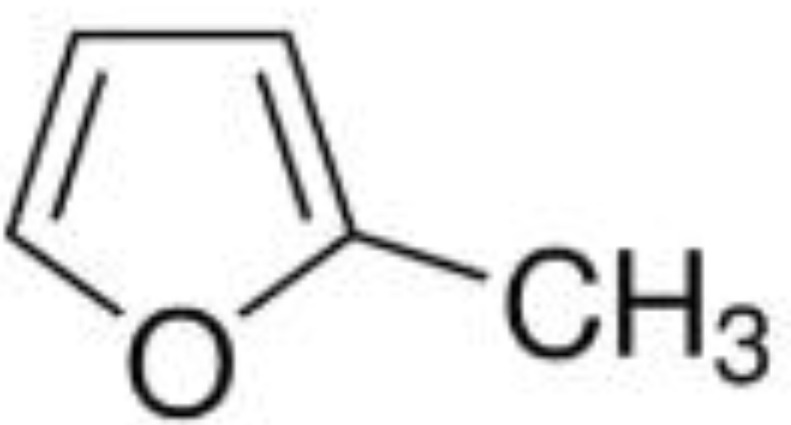	26.26	√	√	√	√	-
Tetrahydrofuran	C_4_H_8_O	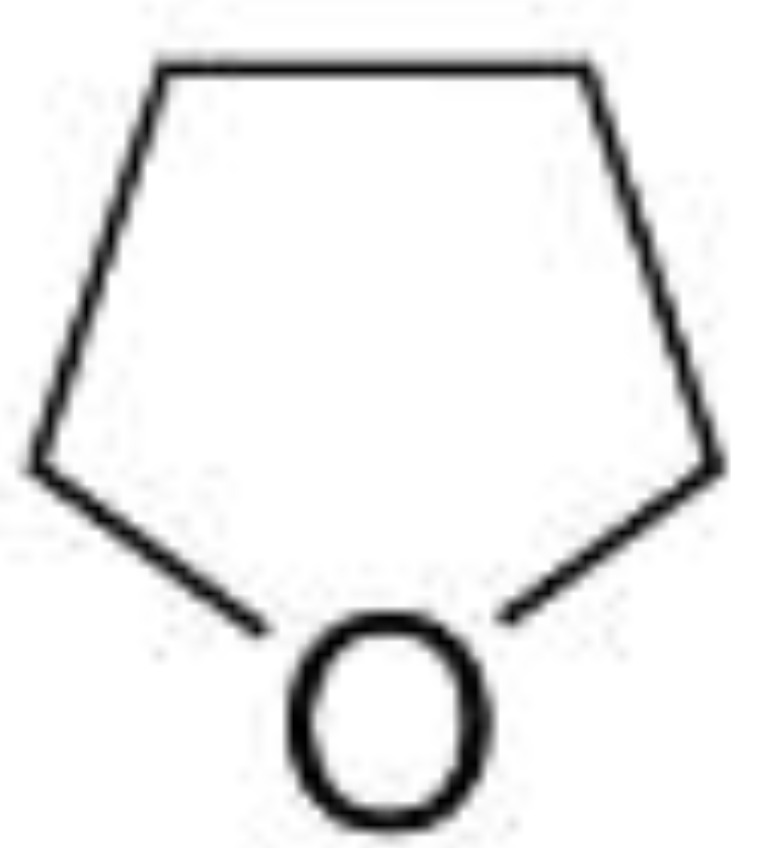	29.17	√	√	-	-	-
2-Methylbutane	C_5_H_12_	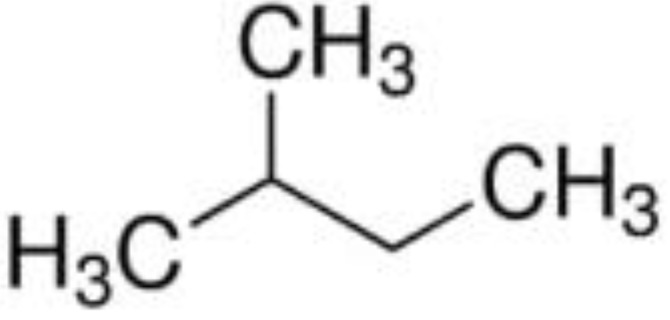	18.34	√	√	√	√	√
Pentane	C_5_H_12_	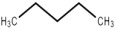	21.33	√	√	√	√	√
Methylcyclopentane	C_6_H_12_	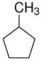	28.91	√	√	-	-	√
Trichloroethane	C_2_H_3_Cl_3_	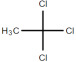	30.37	-	-	-	-	√
Cyclohexane	C_6_H_12_	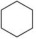	32.50	-	-	-	-	√
Heptane	C_7_H_16_	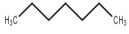	38.87	√	-	-	-	-
1,1-Dichloroethene	C_2_H_2_Cl_2_	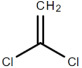	21.33	√	√	√	√	√
Acrylonitrile	C_3_H_3_N	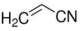	21.33	√	√	√	√	√
Ethane, 1,1-dichloro-	C_2_H_2_Cl_2_	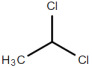	23.69	√	√	√	√	√
Trichloroethylene	C_2_HCl_3_	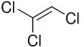	38.87	√	-	-	-	-
Formic acid	CH_2_O_2_	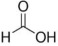	23.69	√	√	√	√	√
Acetic acid	C_2_H_4_O_2_	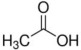	26.26	√	√	√	-	√
Diisopropyl ether	C_6_H_14_O	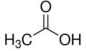	24.94	√	√	√	√	√
Trimethylamine	C_3_H_9_N	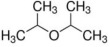	15.90	√	√	√	√	√
Benzene	C_6_H_6_	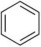	34.12	√	-	-	-	√
Thiophene	C_4_H_4_S	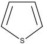	34.12	√	-	-	-	-

Note: - not detected.

## Data Availability

The data are available from the corresponding author.
